# Augmentation index and pulse wave velocity in normotensive versus preeclamptic pregnancies: a prospective case–control study using a new oscillometric method

**DOI:** 10.1080/07853890.2021.2014553

**Published:** 2021-12-22

**Authors:** Christos Anthoulakis, Apostolos Mamopoulos

**Affiliations:** a First Department of Obstetrics & Gynecology, “Papageorgiou" General Hospital, Aristotle University of Thessaloniki, Thessaloniki, Greece; b Third Department of Obstetrics & Gynecology, Hippokration (Ippokrateio) General Hospital, Aristotle University of Thessaloniki, Thessaloniki, Greece

**Keywords:** Cardiovascular diseases, pre-eclampsia, pregnancy complications, pulse wave analysis, vascular stiffness

## Abstract

**Objectives:**

The objective of this study was to investigate whether oscillometric AS measurements are different in pregnant women with and without preeclampsia (PE).

**Study design:**

This was a prospective case–control study in singleton pregnancies that had been diagnosed with PE (*n* = 46) versus normotensive controls (*n* = 46) between 2014 and 2019. In the case group, pregnancies complicated by PE were classified as either early-onset (<34 weeks of gestation) or late-onset (≥34 weeks of gestation) PE and subgroup analysis was performed.

**Main outcome measures:**

Pulse wave velocity (PWV), augmentation index (Alx), and Alx at a heart rate of 75 beats per minute (Alx-75) were measured using a brachial cuff-based automatic oscillometric device (Mobil-O-Graph 24 h PWA).

**Results:**

In pregnancies complicated by PE, in comparison with normotensive pregnancies, there were significant differences in PWV (*p* ˂ .001), and Alx-75 (*p* ˂ .001). In pregnancies complicated by early-onset PE, in comparison with pregnancies complicated by late-onset PE, there were significant differences in PWV (*p* = .006), and Alx-75 (*p* = .009). There was no significant difference in Alx in either of the analyses.

**Conclusions:**

PWV and Alx-75 are higher in pregnancies complicated by PE, in comparison with normotensive pregnancies, as well as in early-onset PE, in comparison with late-onset PE.

**Key messages**
- Pulse wave velocity is higher in pregnancies complicated by preeclampsia.
- Augmentation index at a heart rate of 75 beats per minute is higher in pregnancies complicated by preeclampsia.
- Arterial stiffness assessment is a promising risk-stratification tool for future cardiovascular complications but further studies are required.

## 1. Introduction

Arterial stiffness (AS) is a concept used to describe the rigidity of arterial walls [1]. Epidemiological studies have clearly demonstrated that AS is an independent predictor of cardiovascular (CV) morbidity and mortality in both low- and high-risk non-pregnant populations [2,3]. AS is a marker for increased CV risks such as myocardial infarction, heart failure, chronic kidney disease and all-cause mortality [4–7]. At present, the mechanisms leading to increased AS and the processes underlying the association between increased AS and cardiovascular disease (CVD) remain unclear. Arterial calcification, extracellular matrix degradation and inflammation are likely to contribute to AS [8]. Pulse wave velocity (PWV) and augmentation index (AIx) are well-studied diagnostic tools that are used to assess AS [9]. PWV is considered as a direct marker of AS, whereas AIx is considered as an indirect marker of AS and a direct measure of wave reflection [10]. carotid-femoral PWV (cfPWV) is considered the “gold standard” method for non-invasive measurement of AS [11].

Preeclampsia (PE), a form of pregnancy-induced hypertension, is a leading cause of maternal and perinatal morbidity and mortality, particularly when early-onset [12–15]. Worldwide approximately 5% of pregnancies are complicated by PE [16]. In its severe form, PE may lead to maternal seizure, stroke, intracranial bleeding, coagulopathy, renal failure, pulmonary oedema and death. Foetal consequences may include growth restriction, stillbirth, and complications related to prematurity [17]. The risk of such complications is considerably higher when PE is severe and/or early-onset, leading to preterm birth at less than 37 weeks’ gestation [18–21]. PE is associated with vascular endothelium dysfunction, insulin resistance, hyperlipidaemia, hypercoagulability and inflammation [22–24]. Thus, PE shares many aetiologies with CVD. Women who develop PE are also at increased long-term risk of CVD and stroke in the subsequent decades [12,25]. When compared with patients who did not develop PE, the relative risk (RR) of developing CVD later in life was 2.0 for patients with mild PE and 5.4 for patients with severe PE [26]. Similarly, the RR of death from CVD later in life was 2.1 for patients who had PE at term and 9.5 for patients who were delivered due to PE before 34 weeks’ gestation [27].

PE is associated with increased AS during and after pregnancy, more so than for gestational hypertension [28–30]. In comparison to normotensive pregnancies, carotid-femoral pulse wave velocity (cfPWV) and augmentation index (AIx) were significantly increased in pregnancies complicated by PE [28]. Moreover, normotensive pregnancies compared with pregnancies complicated by PE had (1) in the first trimester; increased AIx-75 (AIx corrected for a heart rate of 75 beats/min), (2) in the second trimester; increased PWV, and (3) in the third trimester; increased PWV and AIx [31]. Additionally, PE is associated with higher postpartum age- and time-adjusted blood pressure (BP) and metabolic indices [32].

Evidence is accumulating that AS measurements can identify women who will later develop PE [33–41]. Elevated pulse pressure, indicating poor arterial compliance, was evident early in pregnancies of women who subsequently developed PE but was not predictive of uncomplicated gestational hypertension [42]. In healthy nulligravid women there is evidence that markers of decreased left ventricular relaxation are associated with increased AS is a family history of myocardial infarction or hypertension [43]. These findings raise the possibility that the diastolic dysfunction and AS observed in the setting of PE are driven by underlying properties present prior to pregnancy and contribute to lifetime CV risk [43]. In women with a history of PE, cfPWV and AIx, assessed at least 3 months postpartum, were increased compared with normotensive pregnancies [44]. However, there is also evidence suggesting that AS can predict the onset of PE [33,38]. Recent studies suggest that maternal predisposition to CVD, manifested as increased PWV and BP, is a pre-pregnancy risk factor for PE [45,46]. This suggests that AS measurements may play a role in the prediction of PE, with AS by itself being an intrinsic part of the increased risk of future CV complications seen in women with a history of PE [27,47].

Several non-invasive methods have been developed to evaluate AS [48]. Applanation tonometers, mechanotransducers, or Doppler probes can be used to measure AS [49–51]. These devices record the transit time of a pulse wave between two arterial sites, such as carotid-brachial or carotid-femoral. Then, the distance between the two locations is divided by the transit time. Doppler probes can be used in radial, brachial, or carotid arteries for local PWV assessments. Important drawbacks of these techniques are that they are time-consuming and require trained operators [52]. In view of simplicity, reliability and reproducibility, there is an increasing interest in oscillometric AS measurements in pregnancies complicated by PE [52].

The objective of this study was to investigate whether oscillometric AS measurements (PWV, Alx, and Alx-75), using 24-h ambulatory blood pressure monitoring, are different in pregnant women with and without PE.

## 2. Materials and methods

### 2.1. Study population

The data for this study were derived from a prospective case–control study for prediction of adverse pregnancy outcomes following diagnosis of PE between July 2014 and August 2020. The women were examined at Ippokrateio General Hospital of Thessaloniki and Papageorgiou General Hospital of Thessaloniki, Greece. We consecutively recruited 46 singleton pregnancies that had been diagnosed with PE and delivered a non-malformed liveborn or stillborn neonate (case group). The normotensive group comprised of 46 singleton pregnancies attending the antenatal clinic, who were matched for maternal age and gestational age with the case group and had uncomplicated pregnancies (control group). With respect to the control group, we excluded women who had a history of hypertensive disorder of pregnancy, intrauterine foetal growth restriction, placental abruption, or used medication that might affect BP. Moreover, in both groups, all potential participants who had the following were excluded from the study: multiple pregnancy; chronic hypertension; coronary artery disease; valvular heart disease; congenital heart disease; heart failure; cardiomyopathy; arrhythmias or conduction disorders on electrocardiography; overt liver disease; cancer; history of alcohol or drug abuse; hematological disease; ongoing infection, systemic inflammatory conditions or any autoimmune disease (such as systemic lupus erythematosus or antiphospholipid antibody syndrome); hyperlipidaemia; pre-existing diabetes mellitus; hypercholesterolaemia; peripheral arterial disease; chronic renal disease; thyroid function abnormalities; significant anaemia (hemoglobin 9 g/dL or less); known psychiatric comorbidities. We excluded pregnancies with aneuploidy or major foetal abnormality.

PE was defined according to the guidelines of the International Society for the Study of Hypertension in Pregnancy [53]. This definition requires a systolic blood pressure of 140 mmHg or higher and/or a diastolic blood pressure of 90 mmHg or higher on at least two occasions four hours apart developing after 20 weeks of gestation in previously normotensive women. Hypertension should be accompanied by proteinuria of 300 mg or more in 24 h or two readings of at least ++ on dipstick analysis of midstream or catheter urine specimens if no 24-hour collection is available. The patients in the PE group were further divided into early-onset and late-onset PE subgroups. Early-onset PE was defined as PE that develops before 34 weeks of gestation, whereas late-onset PE was defined as PE that develops at or after 34 weeks of gestation.

Written informed consent was obtained from the women agreeing to participate in the study, which was approved by the Aristotle University of Thessaloniki Research Ethics Committee and undertaken in accordance with the Declaration of Helsinki. All of the procedures followed were in accordance with institutional guidelines.

### 2.2. Patient characteristics

We recorded maternal characteristics and obstetric history. Patient characteristics recorded included maternal age, weight, height, racial origin (White, Black, South Asian, East Asian and mixed), method of conception (spontaneous or assisted by use of ovulation induction drugs or *in vitro* fertilization), and cigarette smoking during pregnancy. Obstetric history included parity (nulliparous if no previous pregnancy at ≥ 24 weeks), mode of previous delivery (i.e. with or without history of previous caesarean section), previous obstetric complications (i.e. obstetric cholestasis, gestational diabetes mellitus, gestational hypertension, or PE).

### 2.3. Assessment of arterial stiffness parameters

AS was assessed: (1) with respect to the case group, immediately after PE was diagnosed, and (2) with respect to the control group, either between 28 and 33 weeks’ gestation, following serial foetal growth scans in view of increased mean uterine artery pulsatilty index during the 20–23 weeks’ gestation anomaly scan, or between 34 and 36 weeks’ gestation, following the routine foetal growth scan.

24-h non-invasive ambulatory BP monitoring was performed in each study participant, using a Mobile O Graph 24 h PWA (I.E.M. GmbH Stolberg, Germany), for PWV, AIx, and Alx-75 assessment. Mobil-O-Graph is an oscillometric device that records brachial BP and pulse waves and estimates, using a generalized transfer function, Alx as a measure of wave reflection, and PWV as an index of AS. In this study, the AS measurements (PWV, Alx, and Alx-75) were conducted four times per hour between 08:00 and 23:59 and two times per hour between 00:00 and 07:59. Then, the mean ± standard deviation values were automatically calculated for each patient.

### 2.4. Outcome measures

Data on pregnancy outcome were collected from the hospital maternity records. We obtained data for gestational age at delivery, mode of delivery (vaginal delivery or caesarean section), and birth weight. Gestational age was determined by the measurement of foetal crown-rump length at 11–13 weeks or the foetal head circumference at 19–24 weeks. The ultrasound examinations were carried out by examiners who had obtained the Fetal Medicine Foundation Certificate of Competence in ultrasound examination for foetal abnormalities.

### 2.5. Statistical analysis

Data were expressed as median (interquartile range [IQR]) for continuous variables and *n* (%) for categorical variables. Continuous variables were analyzed using the D'Agostino–Pearson test for normality. Normally distributed data were compared using the independent samples *t*-test (assuming equal variances) or *t*-test corrected for unequal variances (Welch test), non-normally distributed data were compared using the Mann–Whitney test (independent samples), and categorical data were compared using *χ*^2^-test or Fisher’s exact test (in case the total number of observations was less than 20). Significance was assumed at 5%. *p*-values were always two-sided.

The statistical software package Medcalc for Windows, Version 12.7.7, 2013 (Medcalc Software, Mariakerke, Belgium) was used for data analyses.

## 3. Results

### 3.1. Normotensive versus preeclamptic pregnancies

Baseline demographic characteristics of the study population are shown in Table 1. There were no significant differences in patient characteristics (racial origin, weight, height, BMI, cigarette smoking), and obstetric history (parity, previous obstetric complications [i.e. obstetric cholestasis, gestational diabetes mellitus, gestational hypertension or PE]), whereas there were significant differences in mode of conception and obstetric history of previous caesarean section between the groups.

**Table 1.** Baseline demographic characteristics (patient characteristics and obstetric history) and pregnancy outcome of the study population.

	Control group, *n* = 46	Case group, *n* = 46	*p* Value
Maternal age in years, median (IQR)	35 (30–40)^a^	36 (27–45)^b^	.252^c^
Maternal weight in kg, median (IQR)	92 (75–109)^b^	94 (63–125)^b^	.173^d^
Maternal height in cm, median (IQR)	168 (160–176)^b^	165 (155–175)^b^	.362^e^
Maternal BMI in kg/m^2^, median (IQR)	32.1 (24.9–39.3)^b^	33.8 (21.9–45.7)^a^	.200^c^
Ethnicity, *n* (% non-Caucasian)	0 (0)	2 (4.3)	.495^f^
Smoking, *n* (%)	6 (13)	12 (26.1)	.188^f^
Method of conception, *n* (% IVF)	4 (8.7)	14 (30.4)	**.016** ^f^
Nulliparity, *n* (%)	24 (52.2)	30 (65.2)	.292^g^
Previous CS, *n* (%)	20 (43.5)	10 (21.7)	**.045** ^g^
Previous PE, *n* (%)	0 (0)	4 (8.7)	.117^f^
Gestational age at examination in weeks, median (IQR)	32 (29–35)^a^	32 (28–36)^b^	.065^c^
Gestational age at delivery in weeks, median (IQR)	38 (37–39)^b^	32 (28–36)^b^	**˂.001** ^c^
Mode of delivery, *n* (% cesarian section)	26 (56.5)	46 (100%)	**<.001** ^g^
Birthweight in g, median (IQR)	3290 (2875–3705)^a^	1695 (840–2550)^b^	**˂.001** ^c^

PE: preeclampsia; *n*: number; IQR: interquartile range; BMI: body mass index; IVF: *in vitro* fertilization; CS: cesarean section. Data are expressed as median (interquartile range [IQR]) for continuous variables and *n* (%) for categorical variables.

^a^Reject normality (D'Agostino–Pearson test for normality).

^b^Accept normality (D'Agostino–Pearson test for normality).

^c^Mann–Whitney test (comparison test).

^d^Welch test (comparison test).

^e^Independent samples *t*-test (comparison test).

^f^Fisher’s exact test (comparison test).

^g^*χ*^2^ test (comparison test).

Data on pregnancy outcome are shown in Table 1. There were significant differences in gestational age at delivery, mode of delivery (vaginal delivery or caesarean section), and birth weight between the groups.

**Figure 1.**
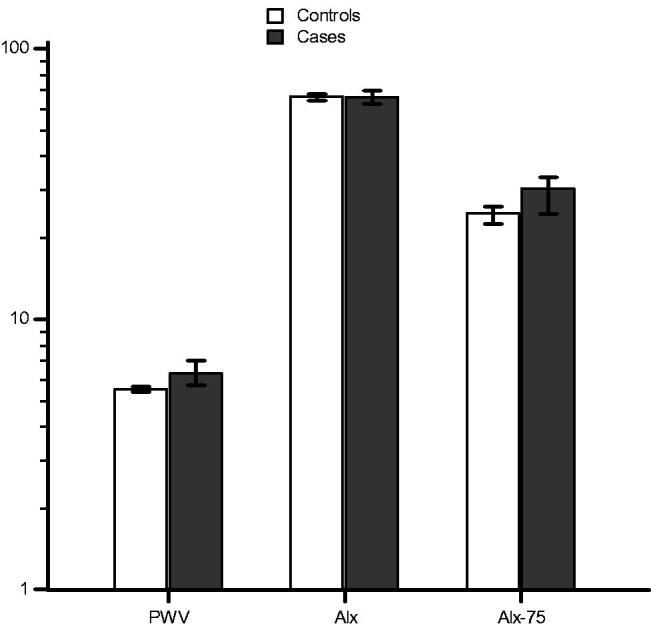
Bar chart for median (error bars represent 25–75 percentiles) of pulse wave velocity (*p*˂.001), augmentation index (*p* = .359), and augmentation index corrected for a heart rate of 75 beats per minute (*p*˂.001), after logarithmic transformation, for case group (pregnancies complicated with preeclampsia) versus control group (normotensive pregnancies).

Data on AS measurements are shown in Table 2. There was no significant difference in Alx, whereas there were significant differences in systolic BP, diastolic BP, PWV, and Alx-75 between the groups (Figure 1).

**Table 2.** Data on AS measurements of the study population.

	Control group, *n* = 46	Case group, *n* = 46	*p* Value
SBP in mmHg, median (IQR)	116 (109–123)^a^	140 (120–160)^b^	**˂.001** ^c^
DBP in mmHg, median (IQR)	75 (67–83)^b^	86 (71–101)^b^	**˂.001** ^d^
PWV in m/s, median (IQR)	5.5 (5.2–5.8)^a^	6.3 (4.9–7.7)^a^	**˂.001** ^c^
Alx in %, median (IQR)	66.6 (63–70.2)^b^	66 (59.1–72.9)^b^	.359^d^
Alx-75 in %, median (IQR)	24.6 (20.9–28.3)^a^	30.5 (20.6–40.4)^b^	**<.001** ^c^

PE: preeclampsia; *n*: number; IQR: interquartile range; SBP: systolic blood pressure; DBP: diastolic blood pressure; PWV: pulse wave velocity; Alx: augmentation index; Alx-75: Alx corrected for a heart rate of 75 beats per minute. Data are expressed as median (interquartile range [IQR]) for continuous variables and *n* (%) for categorical variables.

^a^Reject normality (D'Agostino–Pearson test for normality).

^b^Accept normality (D'Agostino–Pearson test for normality).

^c^Mann–Whitney test (comparison test).

^d^Welch test (comparison test).

### 3.2. Early-onset versus late-onset preeclamptic pregnancies

Baseline demographic characteristics of the study population complicated by early-onset or late-onset preeclampsia are shown in Table 3. There were no significant differences in patient characteristics (height, cigarette smoking), and obstetric history (parity, previous caesarean section, previous obstetric complications [i.e. obstetric cholestasis, gestational diabetes mellitus, gestational hypertension or PE]), whereas there were significant differences in maternal age, weight, BMI, racial origin, and mode of conception between the groups.

**Table 3.** Baseline demographic characteristics (patient characteristics and obstetric history) and pregnancy outcome of the study population complicated by early-onset or late-onset preeclampsia.

	Early-onset PE group, *n* = 36	Late-onset PE group, *n* = 10	*p* Value
Maternal age in years, median (IQR)	36 (31–41)^a^	28 (16.5–39.5)^a^	**.024** ^c^
Maternal weight in kg, median (IQR)	99.5 (63.5–135.5)^a^	87 (69–105)^a^	**.009** ^d^
Maternal height in cm, median (IQR)	164.5 (153.5–175.5)^a^	170 (160.5–179.5)^a^	.735^c^
Maternal BMI in kg/m^2^, median (IQR)	36.9 (24.5–49.3)^b^	31.2 (24.5–37.9)^a^	**.049** ^e^
Ethnicity, *n* (% non-Caucasian)	0 (0)	2 (20)	**.044** ^f^
Smoking, *n* (%)	8 (22.2)	4 (40)	.426^f^
Method of conception, *n* (% IVF)	14 (38.9)	0 (0)	**.020** ^f^
Nulliparity, *n* (%)	26 (72.2)	4 (40)	.145^f^
Previous CS, *n* (%)	8 (22.2)	2 (20)	1.000^f^
Previous PE, *n* (%)	4 (11.1)	0 (0)	.562^f^
Gestational age at examination in weeks, median (IQR)	30 (25–35)^a^	35 (33.5–36.5)^a^	**˂.001** ^d^
Gestational age at delivery in weeks, median (IQR)	30 (26–34)^a^	35 (33.5–36.5)^a^	**˂.001** ^d^
Mode of delivery, *n* (% cesarian section)	36 (100)	10 (100)	–
Birthweight in g, median (IQR)	1410 (625–2195)^b^	2270 (1925–2615)^a^	**˂.001** ^e^

PE: preeclampsia; *n*: number; IQR: interquartile range; BMI: body mass index; IVF: *in vitro* fertilization; CS: cesarean section. Data are expressed as median (interquartile range [IQR]) for continuous variables and *n* (%) for categorical variables.

^a^Accept normality (D'Agostino–Pearson test for normality).

^b^Reject normality (D'Agostino–Pearson test for normality).

^c^Independent samples *t*-test (comparison test).

^d^Welch test (comparison test).

^e^Mann–Whitney test (comparison test).

^f^Fisher’s exact test (comparison test).

Data on pregnancy outcome are shown in Table 3. There was no significant difference in mode of delivery (vaginal delivery or caesarean section), whereas there were significant differences in gestational age at examination, gestational age at delivery and birth weight between the groups.

**Figure 2.**
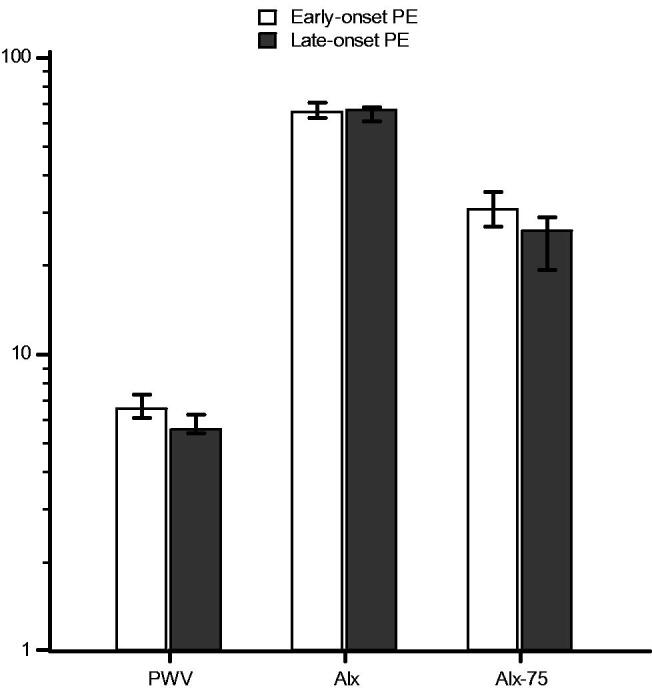
Bar chart for median (error bars represent 25–75 percentiles) of pulse wave velocity (*p* = .006), augmentation index (*p* = .166), and augmentation index corrected for a heart rate of 75 beats per minute (*p* = .005), after logarithmic transformation, for early-onset versus late-onset preeclampsia subgroup.

Data on AS measurements are shown in Table 4. There were no significant differences in diastolic BP, and Alx, whereas there were significant differences in systolic BP, PWV, and Alx-75 between the groups (Figure 2).

**Table 4.** Data on AS measurements of the pregnancies complicated by early-onset or late-onset preeclampsia.

	Early-onset PE group, *n* = 36	Late-onset PE group, *n* = 10	*p* Value
SBP in mmHg, median (IQR)	143.5 (129.5–157.5)^a^	127 (121–133)^a^	**˂.001** ^c^
DBP in mmHg, median (IQR)	87 (73–101)^a^	81 (64.5–97.5)^a^	.075^d^
PWV in m/s, median (IQR)	6.6 (5.4–7.8)^b^	5.6 (4.65–6.55)^a^	**.006** ^e^
Alx in %, median (IQR)	65.4 (57.6–73.2)^a^	66.7 (58–73.4)^a^	.166^d^
Alx-75 in %, median (IQR)	31 (22.7–39.3)^a^	26 (14.5–37.5)^a^	**.009** ^d^

PE: preeclampsia; *n*: number; IQR: interquartile range; SBP: systolic blood pressure; DBP: diastolic blood pressure; PWV: pulse wave velocity; Alx: augmentation index; Alx-75: Alx corrected for a heart rate of 75 beats per minute. Data are expressed as median (interquartile range [IQR]) for continuous variables and n (%) for categorical variables.

^a^Accept normality (D'Agostino–Pearson test for normality).

^b^Reject normality (D'Agostino–Pearson test for normality).

^c^Welch test (comparison test).

^d^Independent samples *t*-test (comparison test).

^e^Mann–Whitney test (comparison test).

## 4. Discussion

The main finding of this study is that, in comparison with normotensive pregnancies, PWV and Alx-75 are different in pregnancies complicated by PE. Subgroup analysis indicates that, in comparison with late-onset PE, PWV, and Alx-75 are different in pregnancies complicated by early-onset PE.

On the one hand, the greatest strength of our study is that, using 24-h ambulatory BP monitoring, it includes a large number of oscillometric AS measurements. Therefore, selection bias is reduced. Moreover, contrary to the existing literature, it performs a subgroup analysis of early-onset versus late-onset PE. On the other hand, the limitations of our study include: (1) the small number of participants and thus the limited amount of data available (especially on late-onset PE); (2) the varying gestational age at AS assessment could affect the differences between subgroups (i.e. early-onset versus late-onset preeclamptic pregnancies); (3) it is unclear whether AS was already increased before the index pregnancy, thus contributing to the development of PE; and (4) the time of the day and study conditions (e.g. abstaining from caffeine) were not standardized. The small study sample limits conclusions to be drawn (especially regarding negative findings) and the possibility to put the results of the current study in relation to previous reports.

Although a significant amount of research has been dedicated in PE prevention, the incidence of PE has been relatively unchanged in the last decades [54]. This could be attributed to the fact that the underlying pathophysiology of PE is not entirely understood [14]. As a consequence, PE has earned the moniker "disease of theories," predominantly because the underlying biological mechanisms linking clinical epidemiologic findings to observed organ dysfunction in PE are far from clear [55]. PE is characterized by a dysfunctional placenta, where impaired maternal spiral artery remodelling can cause intermittent placental hypoxia and ischaemic injury [56]. Identifying the important pathological stages in the progression of PE allows us to evaluate candidate therapeutic options [57]. Three important stages in the pathophysiology are: (1) placental hypoxia and oxidative stress, (2) excess release of anti-angiogenic and pro-inflammatory factors, and (3) widespread systemic endothelial dysfunction and vasoconstriction [57]. There is increasing evidence suggesting that suboptimal trophoblastic invasion leads to an imbalance of angiogenic and antiangiogenic proteins, ultimately causing widespread inflammation and endothelial damage, increased platelet aggregation, and thrombotic events with placental infarcts [14,58–60].

In detail, reduced perfusion of the placenta causes oxidative stress which in turn triggers off release of trophoblast-derived factors which enter the maternal circulation and cause endothelial cell damage in the kidney, liver, brain, and placenta and an exaggerated inflammatory response which underlines many of the changes observed in PE [61]. Placental-derived factors released in response to stress include the anti-angiogenic protein sFLT1 (soluble fms-like tyrosine kinase-1), which is increased in PE [62–64], whereas the circulating concentration of the angiogenic placental growth factor (PlGF) is reduced in PE [63–65]. This angiogenic imbalance results in increased maternal vascular inflammation and generalized endothelial dysfunction.

In contrast to early-onset PE, which is characterized by impaired placentation, in late-onset PE placentation is usually normal [40,55,61]. In women with medical disorders, such as chronic hypertension, there is endothelial dysfunction even before pregnancy [40,55,61]. In such cases, PE can develop in the absence or lower degree of impaired placentation; the pre-existing endothelial dysfunction is further exacerbated by the physiological burden of pregnancy, as normal pregnancies carry a low-grade systemic inflammatory response [40,55,61].

AS per se plays an important role in the increased risk of future cardiovascular complications seen in women with a history of PE. Studies assessing AS between 7 weeks and 2 years postpartum demonstrated significant difference. Hamad et al. [66] found that flow-mediated dilation was decreased in women 1 year after PE pregnancy compared with women with previous normotensive pregnancy. However, studies assessing AS several years after PE pregnancy were inconclusive. Both Lampinen et al. [35] and Ronnback et al. [36] reported non-significant differences between 5 and 9 years postpartum in women with history of PE compared with women with previous normotensive pregnancy. Taking into consideration that Yinon et al. [67] demonstrated differences in AS measurements between early-onset and late-onset PE from 6 to 24 months postpartum, evidence from large longitudinal studies in which BP and AS are determined before the onset of PE with long-term postpartum follow-up would be required to address these issues.

AS measurements have been suggested to be useful in predicting the onset of PE [33,38]. During pregnancy, AIx is reduced in the first two trimesters and then increases in the third trimester, reaching a peak in the postpartum period. Alx as a direct measure of wave reflection is affected by peripheral vascular resistance, heart rate, stroke volume and AS. Therefore, to limit the impact of heart rate, Alx-75 may be a more useful measurement. PWV is affected by age and BP, thus increased PWV may be a direct result of increased BP in pregnancies complicated by PE [68]. Previous studies indicate that, in comparison with tonometric and piezo-electronic methods, the assessment of AS using the oscillometric method is equally reliable in clinical practice [10,50]. Oscillometric AS measurements require no trained operators and the technique is relatively fast, simple, and reproducible.

In the present study, three AS measurements were assessed: (1) PWV, (2) Alx, and (3) Alx-75. In pregnancies complicated by PE, in comparison with normotensive pregnancies, PWV and Alx-75 were significantly different. The differences in mode of conception and obstetric history of previous caesarean section may indicate that preeclamptic pregnancies have a higher baseline CV risk. In pregnancies complicated by early-onset PE, in comparison with late-onset PE, PWV, and Alx-75 were significantly different. Early-onset preeclamptic pregnancies had a higher maternal weight and body mass index (BMI), as well as a much higher *in vitro* fertilization (IVF) conception rate. Maternal obesity (BMI ≥ 35) and complement proteins derived from adipose tissue play an important role in the development of PE [69]. IVF is associated with the onset and progression of PE [70]. Moreover, in view of the relatively low BPs in the late-onset PE group, some degree of the observed differences between pregnancies complicated by PE and normotensive pregnancies could potentially be attributed to the more populous early-onset PE group. There were no significant differences in Alx in either of the analyses. In view of the modest numbers, these results should be interpreted with caution.

Conclusively, women at high risk of PE should be identified as early as possible. In comparison with normotensive pregnancies, PWV and Alx-75 are higher in pregnancies complicated by PE. Oscillometric AS measurements before the onset of PE are required to determine if PWV and Alx-75 are useful in predicting the onset of PE.

The physiological demands of pregnancy act as a maternal stress test that can predict a woman's health in later life. Women with a history of PE are at increased risk of future CV complications. AS independently predicts CV risk and represents a high-priority therapeutic target to ameliorate the global burden of CVD. In comparison with normotensive pregnancies, PWV and AIx-75 are higher in pregnancies complicated by PE. However, in order to detect high-risk pregnancies, AS measurements before the onset of PE are required to determine if PWV and AIx-75 are useful in predicting the onset of PE.

## Data Availability

The data that support the findings of this study are available from the corresponding author, C. A., upon reasonable request.
